# Acute Esophageal Necrosis Following Orthotopic Liver Transplantation

**DOI:** 10.7759/cureus.4090

**Published:** 2019-02-19

**Authors:** Jeffrey A Planchard, Anna F Dikstein, Joseph Koveleskie, Ari Cohen, Grigoriy E Gurvits

**Affiliations:** 1 Anesthesiology, Ochsner Health System, New Orleans, USA; 2 Internal Medicine, State University of New York Downstate Medical Center, Brooklyn, USA; 3 Surgery, Ochsner Health System, New Orleans, USA; 4 Gastroenterology, New York University Langone Medical Center, New York, USA

**Keywords:** acute esophageal necrosis, upper gi bleeding, liver transplantation

## Abstract

Acute esophageal necrosis (AEN) is a rare syndrome characterized by circumferential blackening of the esophageal mucosa extending from the gastroesophageal (GE) junction and affecting variable length of the organ. Its etiology is largely multifactorial including ischemic compromise, massive reflux of gastric secretions, and decreased mucosal defense. Endoscopy is diagnostic. Clinical management requires treatment of underlying condition, nil-per-os restriction, and anti-acids. Esophageal stricture or stenosis may be seen as late complication, managed symptomatically with dilatation. Mortality is high and related to associated medical conditions. We present the first case of AEN following orthotopic liver transplantation.

## Introduction

Acute esophageal necrosis (AEN) or “Black Esophagus” is a rare syndrome characterized by circumferential blackening of the esophageal mucosa [1-2]. This phenomenon starts at the gastroesophageal (GE) junction and may involve variable length of the esophagus [1-6]. The etiology is believed to be from a combination of low-flow state in the celiac-derived blood supply, corrosive tissue injury from gastric reflux, and compromised esophageal protective barrier systems in chronic debilitated state [4]. Associated medical conditions include hemodynamic instability, sepsis, diabetic ketoacidosis, alcohol intoxication, hepatorenal disease, vasculopathy, and malignancy [1, 7]. While mortality in AEN patients has been reported to be as high as 32%, this figure may be misleading as the affected population succumbs to their comorbidities [1, 5, 7-8]. Therefore, AEN is not thought to independently increase mortality [8-10]. We present the first case of AEN following orthotopic liver transplantation.

## Case presentation

A 66-year-old man with alcoholic liver cirrhosis presented for orthotopic liver transplantation with a model for end-stage liver disease (MELD) score 20 on United Network for Organ Sharing (UNOS) waitlist. He had decompensated cirrhosis with hepatic encephalopathy, hypoalbuminemia, hyperbilirubinemia, coagulopathy, thrombocytopenia, portal hypertension, splenomegaly, and ascites requiring frequent paracentesis. He also had secondary restrictive lung disease from a chronic left-sided pleural effusion and pre-existing diabetes mellitus. A pre-transplant esophagogastroduodenoscopy (EGD) showed gastric antral vascular ectasia and Los Angeles Grade B esophagitis.

The patient received a deceased donor liver transplant from a 60-year-old male who died of a cardiac cause. Donor warm time was 26 minutes, cold ischemic time was 373 minutes, and warm ischemic time was 30 minutes. Biopsy of the donor liver showed no significant steatosis, fibrosis, or iron present. The patient remained hemodynamically stable throughout the operation on our typical vasopressor regimen. He was brought intubated to the intensive care unit (ICU) off of vasopressor support.

One hour postoperatively, the patient became hypotensive with mean arterial pressures below 70 mmHg for eight hours requiring escalating doses of vasopressors. After achieving hemodynamic stability, the patient was extubated on POD 0, approximately nine hours after arrival to the ICU. He experienced sustained hyperglycemia requiring an insulin drip for the first 48 hours postoperatively. 

**Figure 1:**
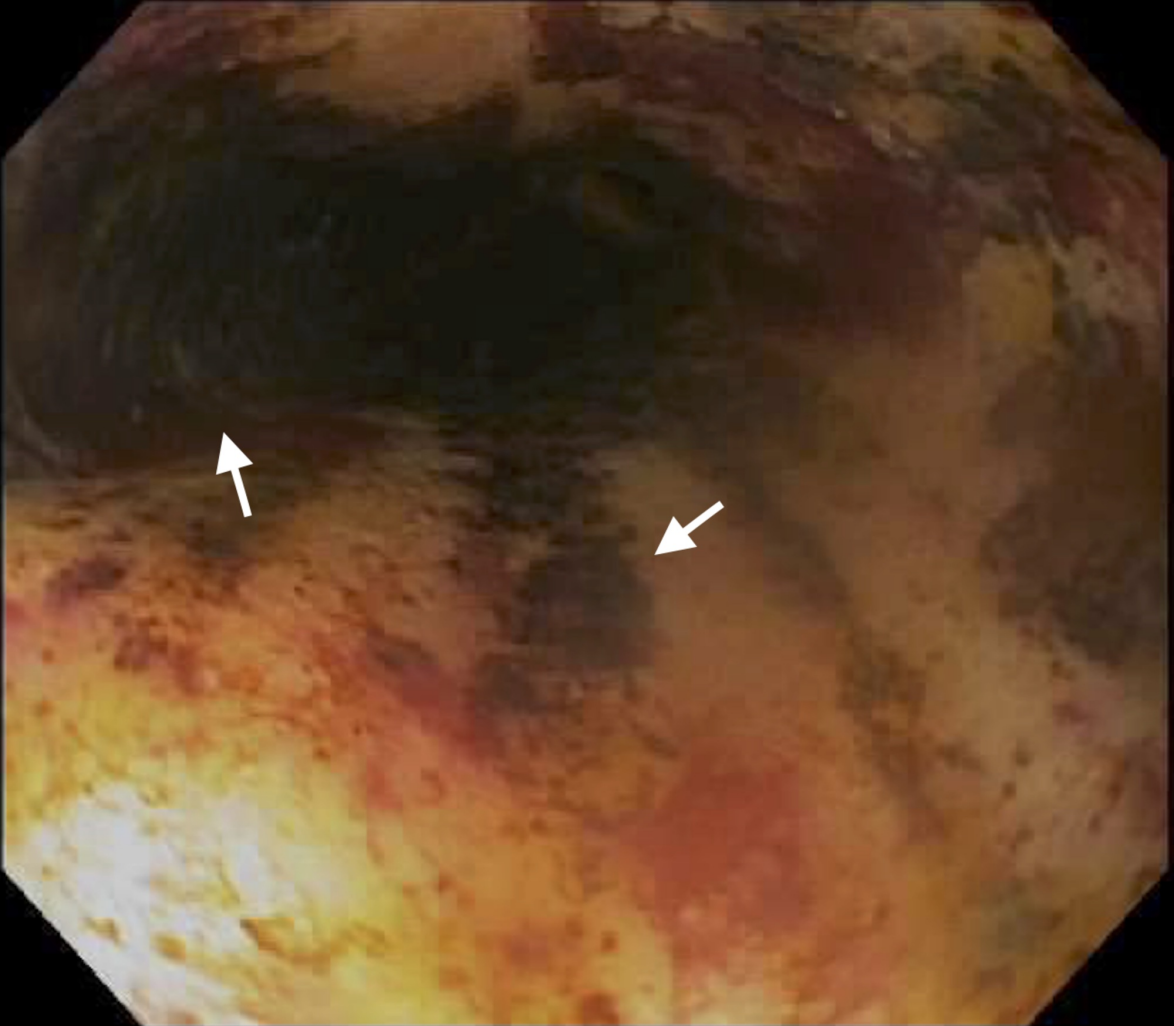
Proximal esophagus – post-operative Day 10. Arrows indicate areas of black-appearing esophageal mucosa.

**Figure 2:**
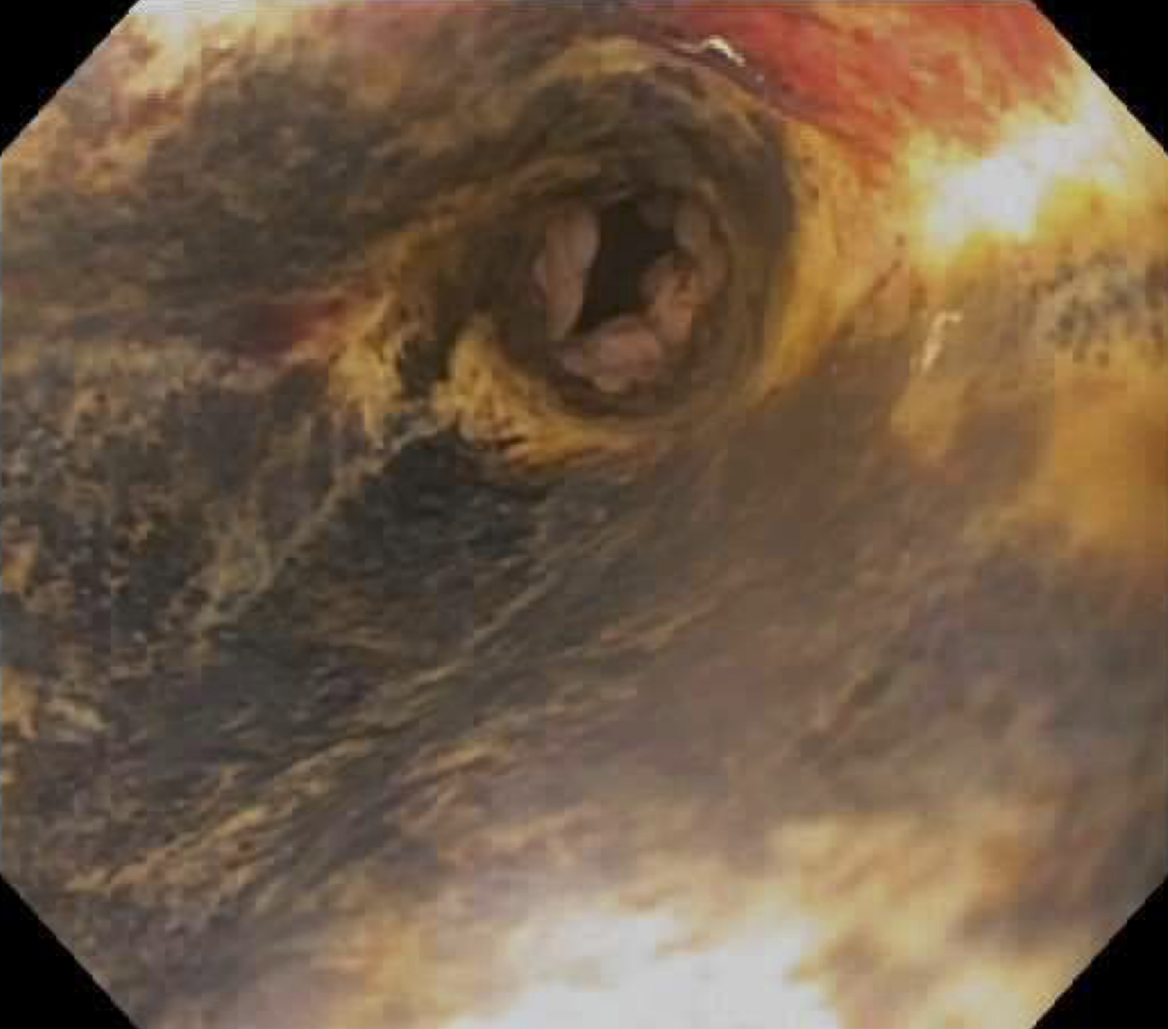
Mid esophagus – post-operative Day 10. Black-appearing esophageal mucosa.

**Figure 3:**
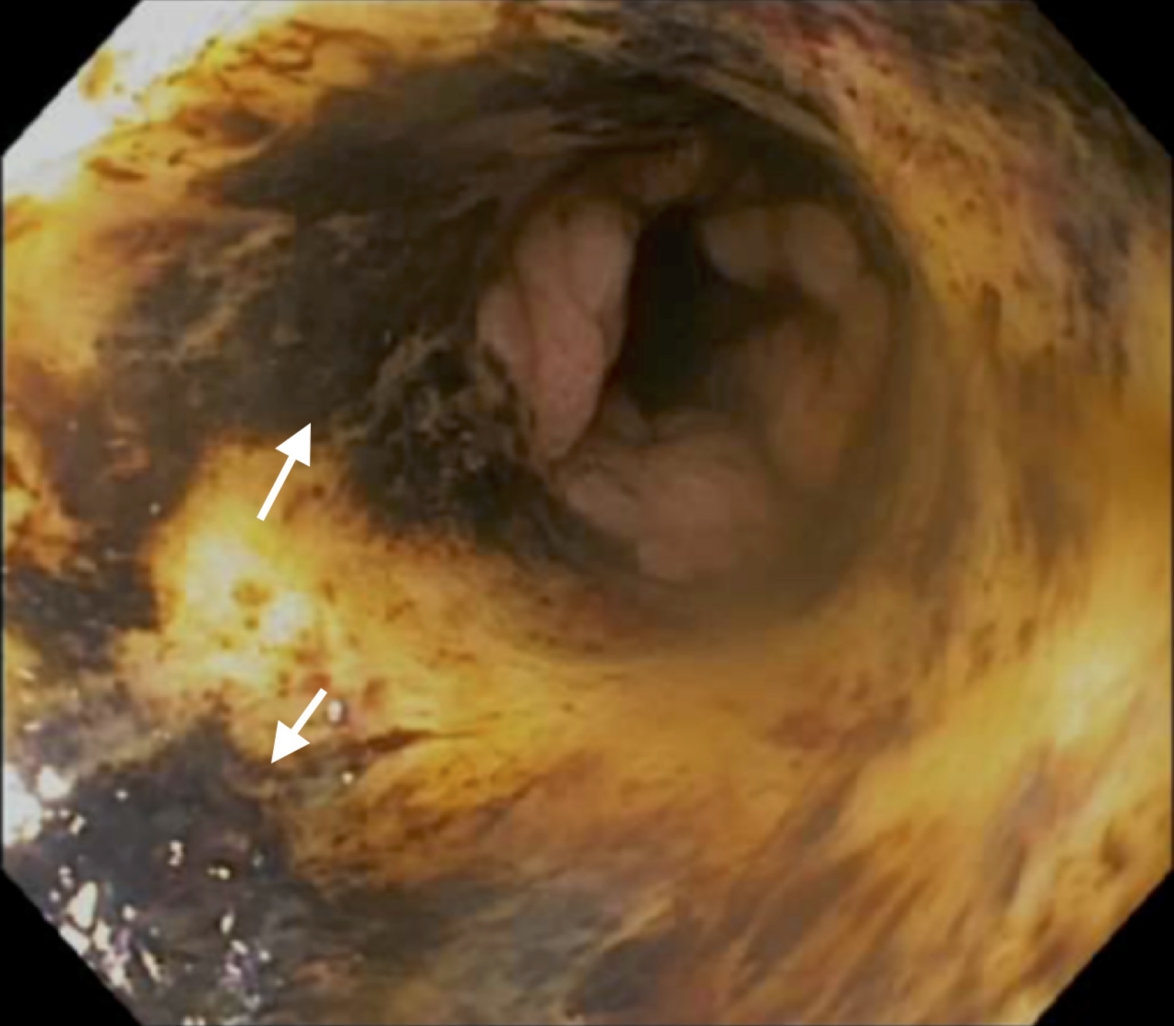
Distal esophagus – post-operative Day 10. Arrows indicate areas of black-appearing esophageal mucosa.

The patient’s diet was advanced in standard fashion, and he exhibited no symptoms between POD’s 0 and 10. On POD 10 a suspected bile leak necessitated an endoscopic retrograde cholangiopancreatography (ERCP). Evaluation revealed a black-appearing esophageal mucosa involving the entire length of the organ, ending at the GE junction (Figures 1-3). No biopsies were taken and the bile duct was stented.

**Figure 4:**
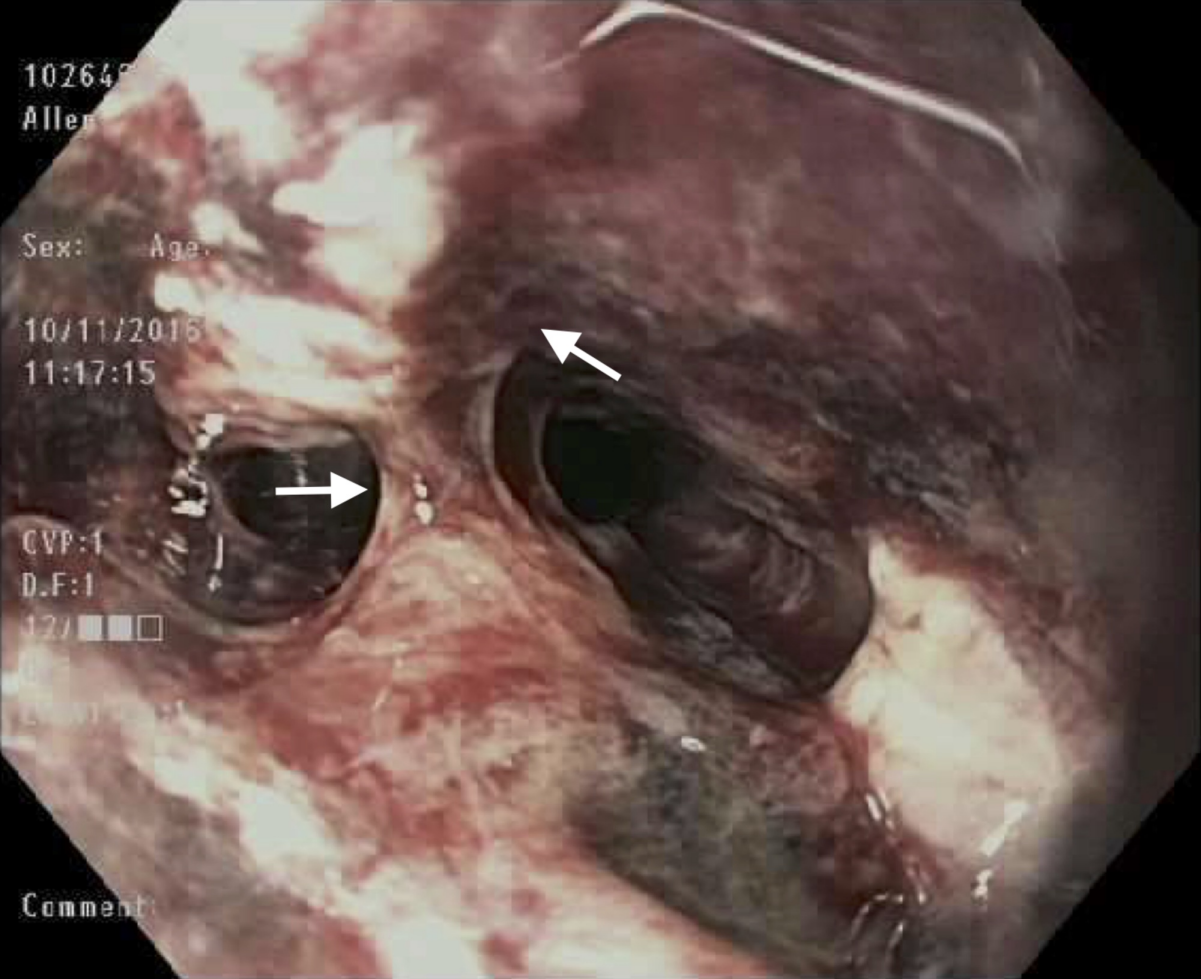
Mid esophagus – post-operative Day 14. Arrows indicate viable pink friable and oozy middle third of the esophagus.

The patient remained nil-per-os, maintained on high dose intravenous proton pump inhibitor therapy, and started on empiric antibiotics, antifungals, and antivirals. A repeat EGD done on POD 14 found viable pink friable and oozy middle third of the esophagus (Figure 4).

**Figure 5:**
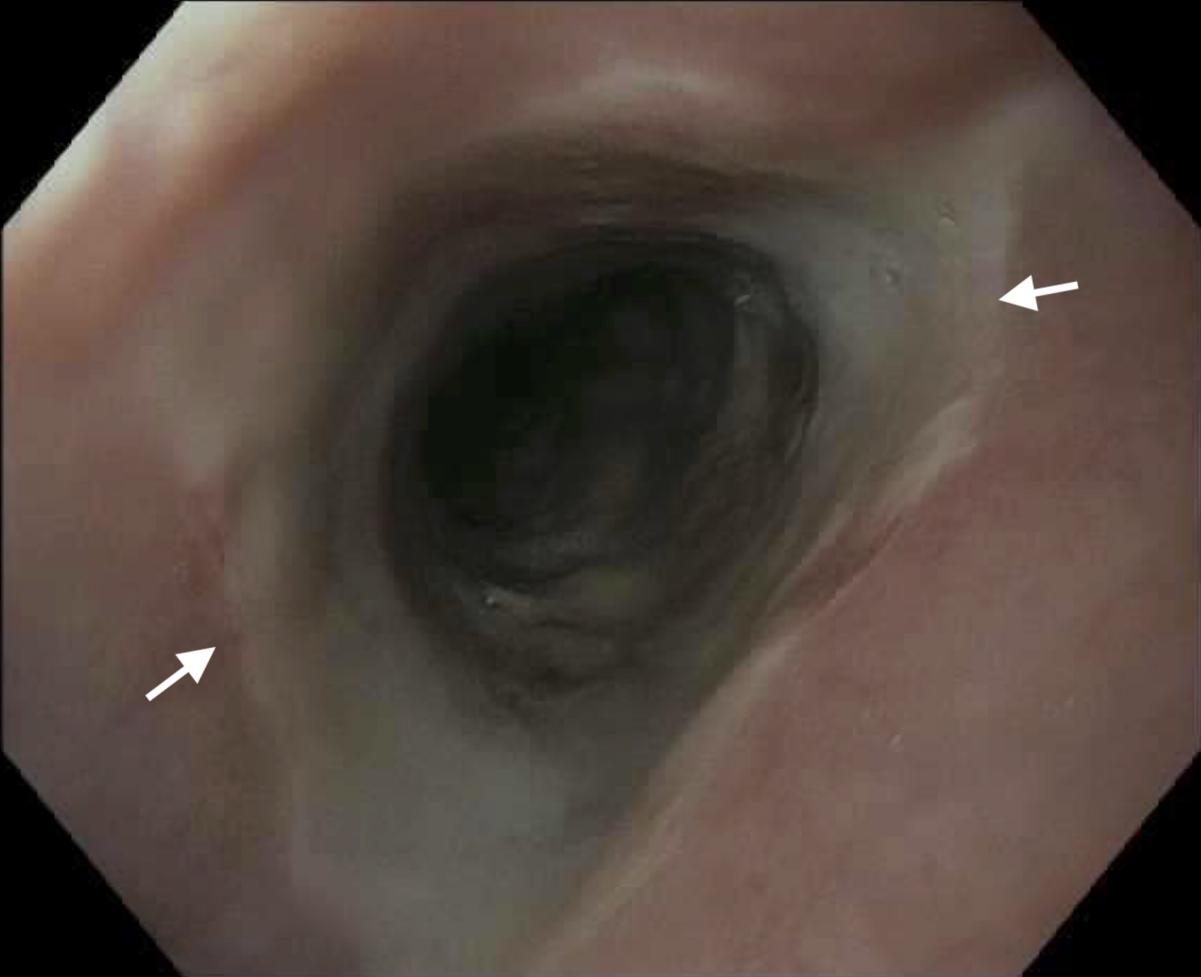
Mid esophagus – post-operative Day 24. Arrows indicate areas of resolution of necrosis from previous endoscopic retrograde cholangiopancreatography (ERCP) (see Figures 1-4).

**Figure 6:**
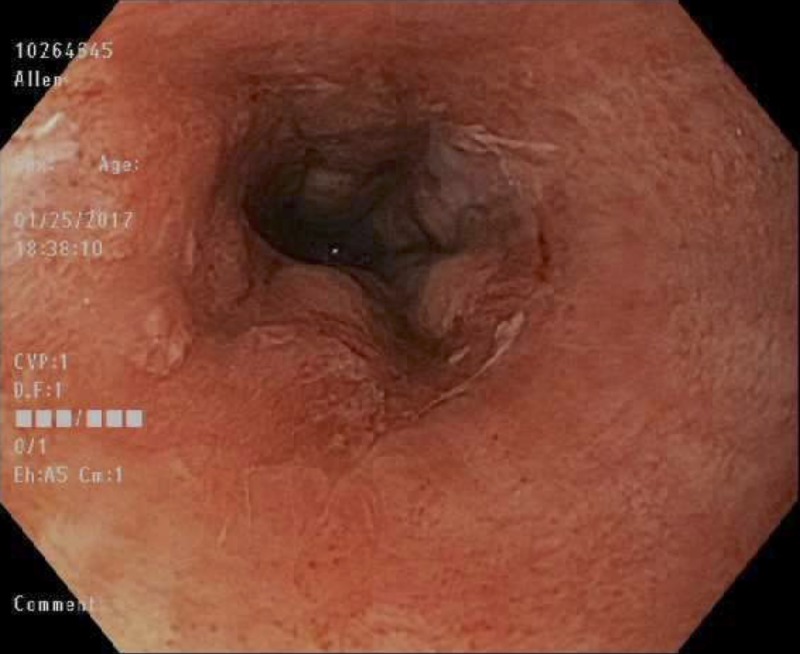
Mid esophagus – four months post-operative. Normal appearing, healed esophagus four months post-operatively.

Despite overall clinical improvement, the patient experienced dysphagia. On POD 23 a repeat EGD showed improvement with resolution of necrosis (Figure 5). By POD 32 the portion of the esophagus previously shown to have diffuse ischemia healed, with a small distal stricture requiring stent placement and removal four months later. This final endoscopy revealed a normal appearing, healed esophagus (Figure 6).

Unfortunately, the patient’s overall health began to deteriorate in his second month of hospitalization. Recurrent pleural effusions necessitated multiple re-intubations and a percutaneous tracheostomy and gastrostomy with repeated bouts of sepsis and shock. Five months after his liver transplantation, the patient expired from sepsis and multi-system organ failure.

## Discussion

While black esophagus was described on post-mortem examinations in the early second half of the 20th century, the AEN was first reported in the gastrointestinal literature by Goldenberg et al. in 1990 and was finally organized in a distinct syndrome by Gurvits et al. in 2007 [7]. Its etiology is multifactorial, a combination of tissue hypoperfusion from low-flow states, corrosive injury from massive reflux, and compromised mucosal defense seen in chronic illnesses. Diagnosis is established at the time of endoscopy with circumferential black-appearing mucosa extending proximally from the GE junction affecting various length of the organ. Histology showing lack of viable epithelium, leukocytic infiltrate, and necrosis is supportive but not required for diagnosis. Associated findings may include duodenal ulcer disease [1]. The blood supply to the esophagus originates from multiple sources including the inferior thyroid, aorta, bronchial, intercostal, and left gastric arteries [6]. It is well established, however, that the distal third of the esophagus has a relatively poor vascular supply relative to other segments [11]. Therefore, transient or prolonged hemodynamic compromise to this area can result in ischemic insult [12] and the universal involvement of the distal third of the esophagus in AEN supports the etiology of a low-flow state [4, 8-9]. Duodenum is more likely to be involved in cases where a greater surface area of the esophagus is affected, likely a reflection of a degree of ischemic and reflux insults resulting from gastric outlet obstruction. 

Prolonged exposure to large volumes of acidic secretions is theorized to severely damage the integrity of the esophageal mucosa and potentially impair blood flow to the area [6-7, 13]. However, despite typical multi-factorial causality, the preponderance of data suggests that celiac hypoperfusion is the primary etiology [6].

Estimations of incidence have varied. A prospective study by Ben Soussan et al. included all 3900 inpatients undergoing endoscopy at a university hospital in 12 months, finding an incidence of 0.2% [5]. Augusto et al. found a similar value of 0.28% after a retrospective analysis of over 10,000 endoscopies [14]. However, Grudell et al. reported an incidence of 0.008% from a retrospective review of 100,000 endoscopies performed from 1963 to 2003 [15]. Moreto et al. (0.0125%) and Lacy et al. (>0.01%) also arrived at low values [5]. Indeed, the overall prevalence is low with only 112 cases reported between 1990 and 2010 [6]. However, the brief clinical course and potential for subclinical presentation make underreporting likely [16-17]. Further, despite a low overall sample size, at-risk patient populations have been identified.

Clinical presentation of AEN varies but typically follows a pattern of upper gastrointestinal hemorrhage in 90% of the cases including melena, hematemesis, or coffee-grounds emesis, although an incidental finding of black esophagus was reported in a patient undergoing percutaneous endoscopic gastrostomy (PEG) placement [1, 7]. Other clues may include epigastric pain, vomiting, and dysphagia. AEN is more commonly seen in males, with a mean age of 67 [1, 4-6]. Comorbid conditions are common and include advanced age, hypoalbuminemia, cardiovascular disease, chronic obstructive pulmonary disease (COPD), hypotension, alcohol abuse, hepatic dysfunction, chronic kidney disease, immunosuppression, hypercoagulable states, malignancy, and diabetes/ hyperglycemia. Up to 75% of the affected patients are malnourished [13].

Management of AEN involves oral restriction, high dose antacids, and correction of underlying medical illness. Repeat endoscopy may reveal rapid esophageal healing. Occurring late in the disease course, esophageal stricture is the condition’s most common long-term complication [1-4, 6]. The incidence of stricture ranges from 10% [2] to 25% [4]. Perforation in full-thickness necrosis is rare and may be seen in approximately 7% and typically presents with rapid clinical deterioration with signs of mediastinitis [1]. AEN is a poor prognostic finding with a significant number of patients succumbing to the advanced medical conditions. However, mortality specifically attributable to AEN is only 6%, usually in the context of significant immunocompromise or esophageal perforations [1]. 

Our case is the first presentation of black esophagus in a newly liver transplanted patient. His demographics fit well with the typical AEN patient seen in the literature: an elderly male with a history of malnutrition (including hypoalbuminemia), liver disease, and alcohol abuse. A hypotensive, ischemic insult occurred 10 days prior to the incidental AEN diagnosis with black esophagus. At the same time, he had uncontrolled hyperglycemia requiring an insulin drip, a condition seen in 90% of cases of AEN [4]. The patient’s lengthy hospital stay allowed our team to observe the entire natural course of the disease. As this patient only became critically ill after his AEN had healed, his scenario remains consistent with the common finding that AEN is independently associated with mortality [3].

## Conclusions

The most notable feature of this case is not its isolated occurrence after liver transplantation, but that it is the first case so documented. Liver transplant patients often carry many of the risk factors commonly associated with AEN: advanced age, male gender, hypoalbuminemia, cardiovascular disease, frequent hypotensive episodes both intra- and post-operatively, alcohol abuse, hepatic dysfunction, chronic kidney disease, and post-operative hyperglycemia (potentiated by graft gluconeogenesis). Because of the considerable overlap between the AEN and liver-transplant patient populations, it is crucial that transplant personnel be made aware of this rare disease. Because of its infrequency, AEN has the capability to be mistaken for far more severe pathology and should be recognized promptly and managed conservatively. As a clinical syndrome confined to the severely ill, AEN also serves as an indirect marker of a patient’s overall prognosis.
